# Inkjet Printing of Long-Range Ordering Two-Dimensional Magnetic Ti_0.8_Co_0.2_O_2_ Film

**DOI:** 10.3390/nano14100834

**Published:** 2024-05-09

**Authors:** Yuntian Du, Pengxiang Zhang

**Affiliations:** State Key Laboratory of Silicate Materials for Architectures, School of Materials Science and Engineering, Wuhan University of Technology, Wuhan 430070, China; duyuntian@whut.edu.cn

**Keywords:** two-dimensional magnetic nanosheets, liquid phase exfoliation, inkjet printing, rheological, long-range ordering

## Abstract

The value of two-dimensional (2D) materials in printed electronics has been gradually explored, and the rheological properties of 2D material dispersions are very different for various printing technologies. Understanding the rheological properties of 2D material dispersions plays a vital role in selecting the optimal manufacturing technology. Inkjet printing is suitable for small nanosheet sizes and low solution viscosity, and it has a significant advantage in developing nanosheet inks because of its masklessness, high efficiency, and high precision. In this work, we selected 2D Ti_0.8_Co_0.2_O_2_ nanosheets, which can be synthesized in large quantities by the liquid phase exfoliation technique; investigated the effects of nanosheet particle size, solution concentration on the rheological properties of the dispersion; and obtained the optimal printing processing method of the dispersion as inkjet printing. The ultrathin Ti_0.8_Co_0.2_O_2_ nanosheet films were prepared by inkjet printing, and their magnetic characteristics were compared with those of Ti_0.8_Co_0.2_O_2_ powder. The films prepared by inkjet printing exhibited long-range ordering, maintaining the nanosheet powders’ paramagnetic characteristics. Our work underscored the potential of inkjet printing as a promising method for fabricating precisely controlled thin films using 2D materials, with applications spanning electronics, sensors, and catalysis.

## 1. Introduction

The development of flexible electronic products and Internet of Things (IoT) has led to a greater demand for miniaturized, flexible, and integrable electronic devices [1]. Printed electronics technology has the advantages of low cost, high productivity, and high adaptability. It, therefore, has a broad application prospect in the fields of flexible electronics, wearable devices, smart packaging, and sensors [2]. These include, among others, inkjet printing [3,4], 3D printing [5], screen printing [6], spin coating [7], spraying [8], and other printing processes [9]. Inkjet printing enables fast, flexible patterning without the need for complex processes. This makes inkjet printing particularly suitable for the development and application of two-dimensional material ink [10,11]. Two-dimensional materials have essential applications in printed electronics due to their mechanical flexibility, transparency, high conductivity, and economy [12]. The preparation of 2D materials by liquid phase exfoliation technology can improve production efficiency and support the raw materials for printed electronics [13,14]. The preparation processes of nanosheets of different sizes have different needs. They bring different effects on the rheological properties of 2D nanosheet colloids. It is important to understand the rheological properties of the solution in the colloidal system of 2D nanosheets in order to choose the appropriate printing method [10].

In recent years, two-dimensional magnetic materials have attracted extensive research interest and become a hot spot in magnetic research. Their magnetic properties are limited and regulated by their two-dimensional structure, and they have potential applications in the fields of magnetic storage, magnetic sensors, and magnetoelectronic devices. Two-dimensional magnetic materials have unique properties in magnetic exchange coupling and spin transport due to their two-dimensional structure, large surface area, and strong surface effects [15]. Secondly, the magnetic properties of 2D magnetic materials can be regulated by external conditions, for example, by applying electric field, strain, etc., to adjust their magnetic properties, which provides new possibilities for their application in magnetic devices. Since the size of 2D magnetic materials is very small, their magnetic properties may be limited. Using 2D nanosheets prepared as thin films to study their magnetic properties to suit various needs may become a major hotspot. Two-dimensional magnetic films can be used to develop more efficient magnetic storage devices, make magnetic field sensors, and develop new types of spintronic devices. With advances in material science, more innovative applications may be developed in the future.

In this work, we prepared Ti_0.8_Co_0.2_O_2_ [16] 2D nanosheets in large and small sizes by two separate methods. The two different sizes of nanosheets were characterized rheologically, respectively, to find a suitable printing method. We found that the viscosity of 2D Ti_0.8_Co_0.2_O_2_ magnetic nanosheets aqueous solution showed obvious shear-thinning behavior, indicating a typical non-Newtonian fluid. The viscosity of about 3.08 cP is relatively low, which is suitable for inkjet printing. For this reason, we set up a composite ink suitable for inkjet printing. After multilayer printing on glass substrates, we obtained long-range ordered ultrathin films and tested their magnetic properties. This will be a new solution for preparing long-range ordered magnetic films using inkjet printing.

## 2. Materials and Methods

Materials. TiO_2_ (99.9%) and tetrabutylammonium hydroxide solution (TBAOH, 40 wt% in H_2_O) were from Sigma-Aldrich Reagent Co., Ltd. (St. Louis, MO, USA); K_2_CO_3_ (99.99%), isopropyl alcohol (IPA, ≥99.9%), HCl (36.5%), and 2-butanol (99%) were procured from Aladdin Reagent Co., Ltd. (Shanghai, China); and CoO (99.9%) and ethanol (AR, ≥99.7%) were procured from Sinopharm Chemical Reagent Co., Ltd. (Shanghai, China). All chemical reagents were commercially available and used as received. Milli-Q water (18.2 MΩ cm, 25 °C) was used for the whole experiment.

Synthesis of Ti_0.8_Co_0.2_O_2_ nanosheets. Ti_0.8_Co_0.2_O_2_ nanosheet preparation method has been reported in the literature, and the actual synthesis process was adjusted as follows: using TiO_2_, K_2_CO_3_, and CoO as raw materials, they were mixed, warmed up to 900 °C, and pre-cooked for 1 h. After pre-sintering and cooling to room temperature, milling and calcination were performed for 20 h to make K_0.8_Ti_1.6_Co_0.4_O_4_ precursor. After weighing 3 g of the layered compound K_0.8_Ti_1.6_Co_0.4_O_4_, adding hydrochloric acid, and shaking the treatment, the product of the reaction was filtered, washed with water to neutrality, and subsequently dried at room temperature, thus obtaining the protonated layered compound H_0.8_Ti_1.6_Co_0.4_O_4_·*n*H_2_O powder. Then, 1 g of protonated compound H_0.8_Ti_1.6_Co_0.4_O_4_·*n*H_2_O was weighed and added to 250 mL of tetrabutylammonium hydroxide (TBAOH) solution. The obtained mixed solution was placed in a conical flask and sonicated in an ultrasonic 2D material stripper at 432 W power for 30 min to obtain the exfoliated monolayer Ti_0.8_Co_0.2_O_2_ nanosheet solution. The solution was centrifuged at 4000 r/min for 15 min, and the upper nanosheet solution was collected and cold-dried to obtain flocculated monolayer Ti_0.8_Co_0.2_O_2_ nanosheets.

Ink Preparation. The varnish was prepared in the ratio of isopropanol:water:2-butanol = 9:4:1, and flocculated nanosheets were added at a concentration of 1 mg/mL, and the IPA-based Ti_0.8_Co_0.2_O_2_ ink was obtained after ultrasonication.

Inkjet printing. The patterns and magnetic films were printed using a DoD inkjet printer (Scientific 3, Shanghai Mifang Electronic Technology Co., Ltd., Shanghai, China) equipped with a 16-nozzle cartridge with a nozzle diameter of 30 μm and typical droplet volume of 10 pL (Dimatix11610, Fujifilm Dimatix, Santa Clara, CA, USA).

Characterization. X-ray diffractometry (SmartLab SE, Rigaku, Cu Ka radiation, λ = 1.54056 Å) was performed to explore the compositions and crystal structures of the samples. The morphology and microstructures of nanosheets and printed samples were characterized by scanning electron microscopy (SEM, JSM-7610F Plus, JEOL Ltd., Tokyo, Japan), transmission electron microscopy (TEM, JEM-F200, JEOL Ltd., Tokyo, Japan), and atomic force microscopy (AFM, Asylum Research Cypher ES, Santa Barbara, CA, USA). X-ray photoelectron spectroscopy tested the atomic valence states (XPS, ESCALAB 250Xi, Thermo Fisher Scientific, Waltham, MA, USA). Rheological characteristics were determined by a rotational rheometer (Kinexus Pro+, NETZSCH, Selb, Germany). A QBZY-2 liquid surface/interface tensiometer was used to measure the surface tension of the inks at room temperature. The zeta (*ζ*) potential of inks was collected by a zeta potential analyzer (Malvern Zetasizer Nano ZS90, Malvern Instruments Ltd., Westborough, MA, USA). Magnetic properties of the nanosheet powders and thin films were tested using a superconducting quantum interferometric magnetic measurement system (MPMS, MPMS-3, Quantum Design, San Diego, CA, USA) at a magnetic field strength of 2 T and a temperature of 300 K.

## 3. Results

### 3.1. Synthesis of Ti_0.8_Co_0.2_O_2_ Nanosheets

**Figure 1:**
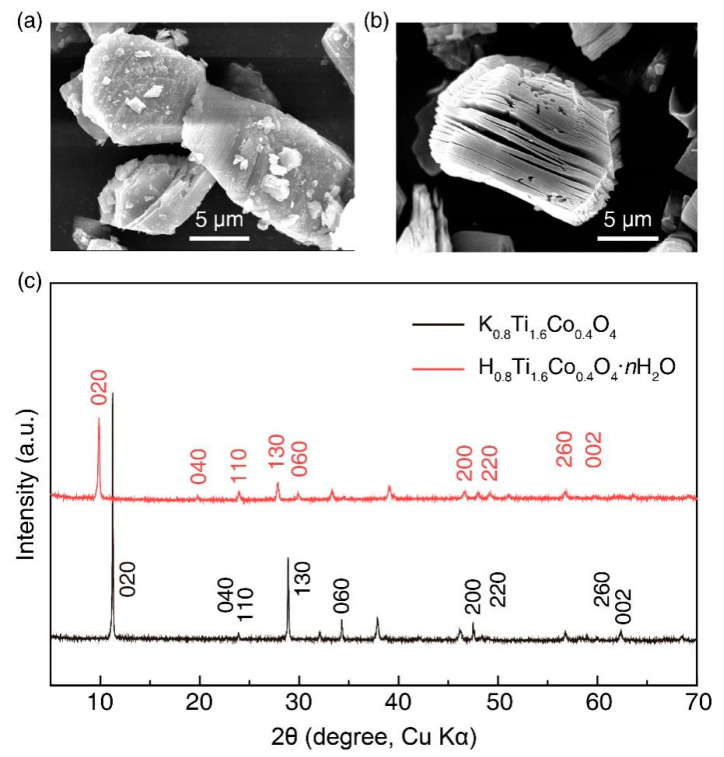
Synthesis of Ti_0.8_Co_0.2_O_2_ nanosheets. (**a**) SEM images of K_0.8_Ti_1.6_Co_0.4_O_4_ and (**b**) H_0.8_Ti_1.6_Co_0.4_O_4_·*n*H_2_O powders. (**c**) XRD diagrams of K_0.8_Ti_1.6_Co_0.4_O_4_ and H_0.8_Ti_1.6_Co_0.4_O_4_·*n*H_2_O powders.

The layered phase K_0.8_Ti_1.6_Co_0.4_O_4_ and proton phase H_0.8_Ti_1.6_Co_0.4_O_4_·*n*H_2_O powders were synthesized by solid phase reaction as described in the experimental section [17]. Figure 1a shows the SEM image of the layered compound K_0.8_Ti_1.6_Co_0.4_O_4_. It can be seen that most of the powder samples were distributed in flat lumps and slightly swollen, with powder sizes between 10 μm and 15 μm, and the lamellar structure was apparent. After mixing hydrochloric acid and K_0.8_Ti_1.6_Co_0.4_O_4_ sufficiently, H^+^ in HCL replaced the alkali metal ion K^+^ in the lamellar compound. As the reaction proceeded, H_2_O molecules also entered the middle of the lamellae of the layered compounds, and the reaction product, proton phase H_0.8_Ti_1.6_Co_0.4_O_4_·*n*H_2_O, was finally obtained. The SEM image was shown in Figure 1b, and a very obvious layered structure can be observed. Figure 1c compares the XRD diagrams of the layered compounds K_0.8_Ti_1.6_Co_0.4_O_4_ and H_0.8_Ti_1.6_Co_0.4_O_4_·*n*H_2_O, respectively, in which the same crystallographic indices are labelled. It can be found that the peaks of the H-phase compounds were shifted to the left compared with those of the lamellar phase compounds, which indicated that the acid-exchange reaction was complete, which made the interlayer spacing larger. The combination of the SEM images together proved that the proton phase compounds were successfully synthesized.

### 3.2. Characterization of Ti_0.8_Co_0.2_O_2_ Nanosheets

**Figure 2:**
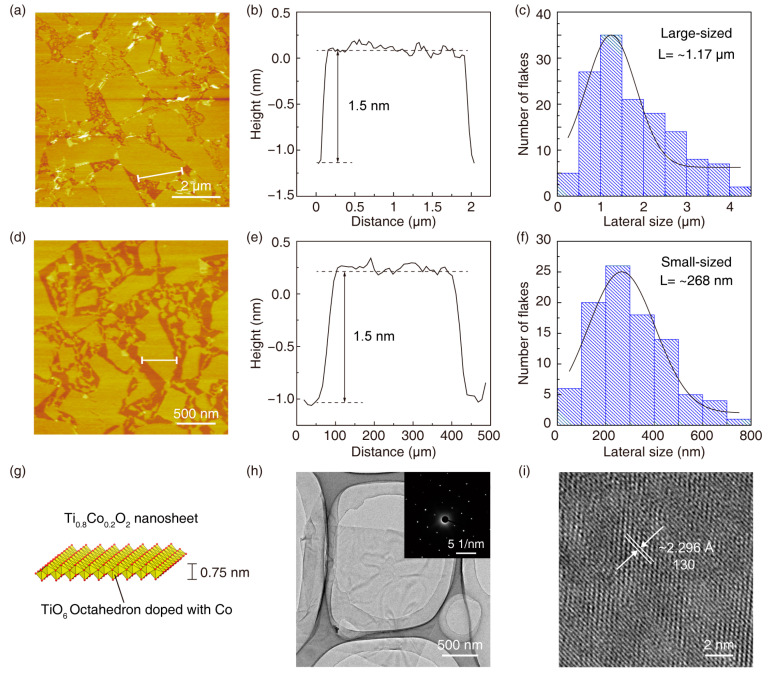
Characterization of Ti_0.8_Co_0.2_O_2_ nanosheets. AFM images of (**a**) small- and (**d**) large-size nanosheets. Height profiles across the individual (**b**) small- and (**e**) large-sized nanosheets. (**c**) Lateral size distribution of (**c**) small- and (**f**) large-sized nanosheets. (**g**) Crystal structure of Ti_0.8_Co_0.2_O_2_ nanosheets. (**h**) TEM and (**i**) HRTEM images of Ti_0.8_Co_0.2_O_2_ nanosheet.

In order to study the printing rheological properties of Ti_0.8_Co_0.2_O_2_, we purposely prepared Ti_0.8_Co_0.2_O_2_ nanosheets in large and small sizes using two different methods [18]. By manual shaking with a long reaction time, we prepared large-size Ti_0.8_Co_0.2_O_2_ nanosheets with a size larger than 2 μm (Figure 2a–c). Atomic force microscopy (AFM) images showed that the Ti_0.8_Co_0.2_O_2_ nanosheets had a typical two-dimensional morphology with a thickness of about 1.2 nm and a lateral size of about 2 μm. The small-sized Ti_0.8_Co_0.2_O_2_ nanosheets prepared by liquid phase exfoliation also had a typical two-dimensional morphology with a thickness of about 1.2 nm and a lateral size of about 500 nm (Figure 2d–f). Figure 2g shows the crystal structure of the Ti_0.8_Co_0.2_O_2_ nanosheet. Figure 2h shows a low-magnification transmission electron microscopy (TEM) image of a single Ti_0.8_Co_0.2_O_2_ nanosheet, which showed the obvious ultrathin features typical of a two-dimensional crystal, and the diffraction spots of the single crystal were detected by the electron diffraction image. A clear lattice diffraction pattern can be observed in Figure 2i. These results indicated that the exfoliated Ti_0.8_Co_0.2_O_2_ nanosheets retained the atomic arrangement of the host layer in the starting lamellar structure.

### 3.3. Rheological Properties of Ti_0.8_Co_0.2_O_2_ Nanosheet Inks

**Figure 3:**
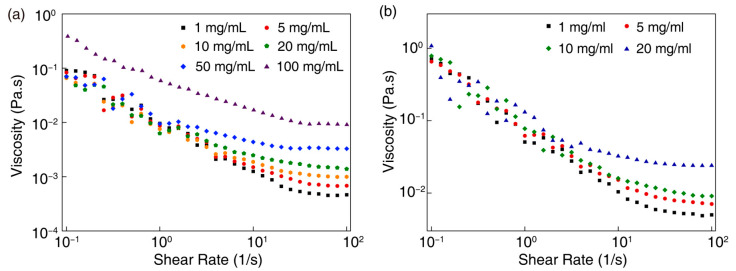
(**a**) Viscosity versus shear rate for (**a**) small- and (**b**) large-sized nanosheets.

We have further explored the rheological properties of small- and large-sized Ti_0.8_Co_0.2_O_2_ nanosheets. The rheological properties of the aqueous solutions of nanosheets have been determined by rotational rheometer. Figure 3 shows the relationship between the viscosity of Ti_0.8_Co_0.2_O_2_ nanosheet inks and the shear rate variation. The viscosity law was not too evident at low shear rates, and the higher the concentration of the nanosheet inks at high shear rates, the greater the fluid’s viscosity. Figure 3 suggests that all the samples showed the phenomenon of shear thinning, which is consistent with pseudoplastic fluid behavior [2]. At higher shear rates, the shear stress of the pseudoplastic fluid decreases, and the fluid requires less force to keep flowing. Pseudoplastic behavior was essential for ink formulation because, in this case, the pigments in ink were more easily dispersed under pressure [19]. Pseudoplastic behavior also facilitates the ink transfer in the component, thus flowing through the press. When the ink reaches the piezo printheads, the viscosity was reduced due to squeezing by the chamber, and the droplets were easier to eject, which facilitates inkjet printing. However, the ink was at a low shear rate once printed on the substrate. It became more viscous, so it did not spread excessively, preventing splattering and contributing to improved print resolution. Pseudoplastic fluids had controllable viscosity and printability and can be adapted to different printing processes. Figure 3a,b, Figures S2 and S3 show that the viscosity difference between small-size Ti_0.8_Co_0.2_O_2_ nanosheets and large-size Ti_0.8_Co_0.2_O_2_ nanosheets was insignificant at low and high concentrations. They did not have the viscoelastic modulus of high-viscosity fluids, which is unsuitable for screen printing, 3D printing, and other printing methods with high viscosity requirements, so we chose the inkjet printing method to print them [20].

### 3.4. Characterization of Inkjet-Printed Ti_0.8_Co_0.2_O_2_ Nanosheet Films

Inkjet printing has been widely used as a printing method for efficient, large-scale manufacturing of electronic devices because of its advantages of masklessness, high precision, and suitability for various substrates [21]. Ultrasonic exfoliation technology has been well integrated with inkjet printing as a means of mass production of 2D nanosheets, and N-methylpyrrolidone (NMP) [22] and N, N-dimethylformamide (DMF) [23] have been widely used as common solvents for liquid exfoliation and ink formulations. However, their high boiling point and toxicity make removing these organic solvents from printed electronic devices more difficult. In our case, Ti_0.8_Co_0.2_O_2_ nanoflakes were directly exfoliated in water, so water was chosen as a solvent for preparing inks with good dispersion and stability. Water, isopropanol, and isobutanol were chosen as solvents due to their low toxicity, low boiling point and low cost. In this formulation, isopropanol was used as the primary solvent because it has good printability, i.e., suitable viscosity and surface tension. Due to the hydrophilic nature of the nanoflakes, water was used to further disperse and stabilize the nanosheets. 2-butanol inhibits the coffee effect mainly through the Marangoni effect. The fluidic properties can be estimated qualitatively using the inverse Ohnesorge (*Oh*) number [24]: *Z* = *Oh*^−1^ = (*γρa*)^1/2^/*η*, where *η* denotes the ink viscosity, *γ* denotes the surface tension, *ρ* denotes the density, and *a* denotes the nozzle diameter. It is known that a value of *Z* between 1 and 14 indicates a stable inkjet, where *Z* < 1 indicates a non-jetted ink and *Z* > 14 indicates an ink that jots satellite droplets. For 1 mg/mL ink, the nozzle diameter was 20 μm, the surface tension was 23.56 mN/m, the viscosity was 3.08 cP, and the constant *Z* was 6.50, which met the requirements for printing.

**Figure 4:**
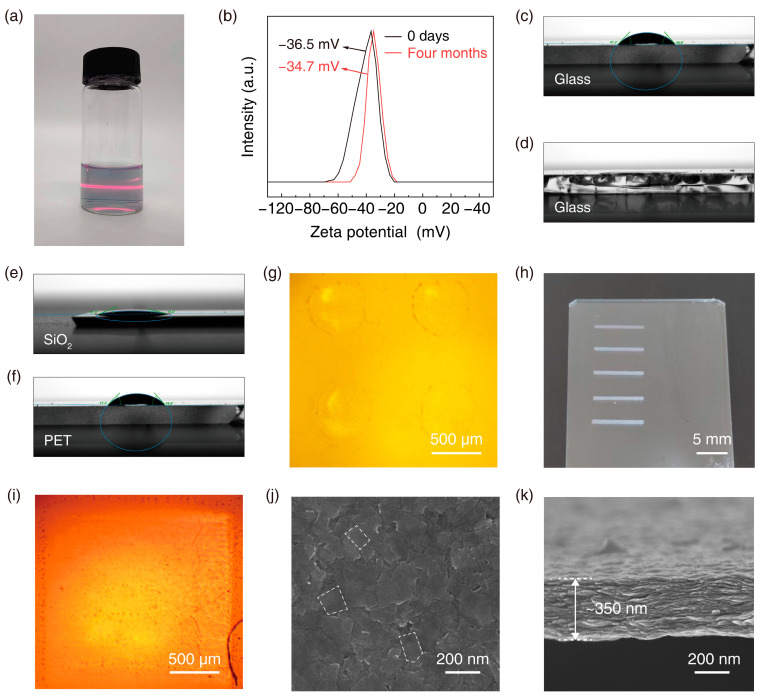
(**a**) The optical photographs of Ti_0.8_Co_0.2_O_2_ inks. (**b**) The zeta potential (ζ) of Ti_0.8_Co_0.2_O_2_ inks. The contact angle of (**c**) Ti_0.8_Co_0.2_O_2_ nanosheet aqueous solution and (**d**) ink on a glass substrate. The contact angle images of Ti_0.8_Co_0.2_O_2_ nanosheet inks on (**e**) SiO_2_ and (**f**) PET substrate. (**g**–**i**) The optical photographs of the printed dots, lines, and planes on a glass substrate. The (**j**) surface and (**k**) cross-section SEM images of the printed Ti_0.8_Co_0.2_O_2_ films on a glass substrate.

Figure 4a shows an optical photograph of the water-based Ti_0.8_Co_0.2_O_2_ ink. An apparent Tyndall effect was observed, indicating that the Ti_0.8_Co_0.2_O_2_ nanosheets were well dispersed in the studied inks and showed good stability over a few months without aggregation of the nanosheets, which was demonstrated by the high zeta potential of Figure 4b. The contact angle of the aqueous solution (Figure 4c) and the hybrid ink (Figure 4d) on the glass substrate was tested, and it can be found that the aqueous solution has a large contact angle on the glass substrate, which was unfavorable for the spreading of the droplets. In contrast, the hybrid ink spread well and was suitable for printing. At the same time, we conducted contact angle tests of the hybrid ink (Figure 4e,f) and its control aqueous solution (Figure S3a,b) on both silicon and PET substrates. It was confirmed that our hybrid ink had a better spreading effect. The configured composite inks were inkjet printed on glass substrates, and Figure 4g–i shows the AFM images printed on glass substrates. It can be found that the printed single layer of dot droplets can be spread out entirely with more apparent print lines. In the printed 20-layer square film stacked on the glass substrate, none of the printed images had an obvious coffee ring effect, which confirmed the reliability of the print quality due to the Marangoni effect [25]. Figure 4k shows a cross-section image of the printed 50-layer film. The thickness of the printed 50-layer film was 350 nm, confirming that the printed film can be stacked with ultrathin thickness. Figure 4j shows the surface image of the printed 50-layer film, where the alignment of the nanosheets on the surface can be observed. The two-dimensional nature of the nanosheets enabled them to be laid down on the substrate in a layered manner, resulting in their long-range ordered arrangement [26]. This will facilitate further research on the properties of 2D materials and the construction of electronic devices with even better performance.

### 3.5. Magnetic Properties of Inkjet-Printed Ti_0.8_Co_0.2_O_2_ Nanosheet Films

**Figure 5:**
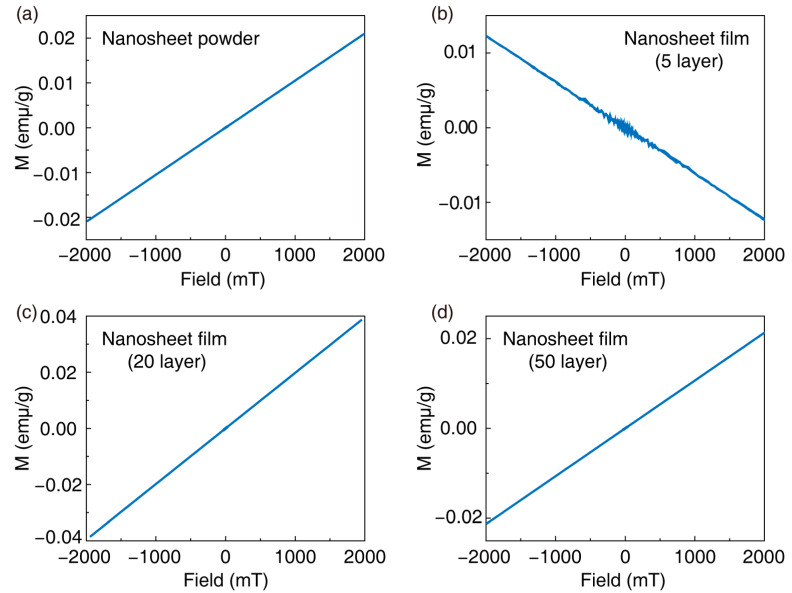
(**a**) M-H curves of Ti_0.8_Co_0.2_O_2_ nanosheet powders. M-H curves for printing (**b**) 5-, (**c**) 20-, and (**d**) 50-layer films.

To investigate the influence of film thickness on the performance of inkjet-printed films, we tested the magnetic properties of films printed with 5, 20, and 50 layers. Before this, we tested magnetic Ti_0.8_Co_0.2_O_2_ nanosheet powders, which exhibited paramagnetism, as shown in Figure 5a. It was worth noting that the reported magnetic behavior of Ti_0.8_Co_0.2_O_2_ nanosheets so far pertained to ultrathin films [12,16,27]. These films possessed extremely high density and orientation, with insulating layers between each layer to maintain isolation and avoid the anisotropic behavior of spin electrons in different orientations, which may affect the magnetic properties of ultrathin films. Due to Ti_0.8_Co_0.2_O_2_ nanosheets being composed entirely of surface atoms arranged in a two-dimensional array, the surface Co spin and its local ferromagnetic coupling were very strong. However, in magnetic powders, the orientation of nanosheets was non-uniform, leading to paramagnetism in Ti_0.8_Co_0.2_O_2_ nanosheet powders, as shown in Figure 5a. We tested the magnetic properties of printed 5-layer, 20-layer, and 50-layer films at room temperature, as shown in Figure 5b–d. The results showed that the 5-layer and 20-layer printed films exhibited the same paramagnetic behavior as the powder, indicating that the magnetic behavior identical to the nanosheet powder was maintained even in long-range ordered printed films. However, the reason the printed films did not exhibit significant ferromagnetism may be due to interference from the quartz substrate during testing and poorer orientation caused by insufficient stacking of nanosheets when the number of layers was low. The 50-layer printed films displayed pronounced diamagnetic behavior, indicating that as the thickness of the film increased, Ti_0.8_Co_0.2_O_2_ nanosheets struggled to maintain uniform orientation. Although significant long-range ordered behavior was observed, it did not contribute to maintaining the magnetic properties of the nanosheets in the case of the 50-layer film. Research on magnetic films requires minimizing film thickness while meeting densification requirements. As an efficient printing method without masks, inkjet printing has been successfully demonstrated in our work to have the capability to print dense, long-range ordered, ultrathin films. However, the densification of magnetic films still needs improvement. In future work, utilizing large-sized magnetic nanosheets and printing techniques with good film-forming orientation, such as spin coating and blade coating, may further enhance the magnetic performance of printed films.

## 4. Conclusions

In summary, we successfully synthesized small- and large-sized monolayer Ti_0.8_Co_0.2_O_2_ nanosheets via liquid phase exfoliation. These nanosheets were subsequently formulated into ink for rheological analysis, revealing a consistent shear-thinning behavior characteristic of typical pseudoplastic fluids. Building upon this, we further developed inkjet-printable Ti_0.8_Co_0.2_O_2_ nanosheet ink, demonstrating excellent stability and dispersibility for over 4 months. This ink facilitated the successful inkjet printing of Ti_0.8_Co_0.2_O_2_ nanosheet films, which exhibited dense and distinct long-range ordering behavior when printed on glass substrates. These printed films displayed the same pronounced paramagnetic behavior as the nanosheet powders. However, they did not exhibit significant ferromagnetic properties, possibly due to insufficient density in the inkjet-printed films and the small size of the Ti_0.8_Co_0.2_O_2_ nanosheets, leading to increased penetration pathways. Utilizing larger-sized nanosheets and employing printing techniques with improved film-forming orientation may further enhance the magnetic properties of the printed films. Our work significantly contributes to the preparation and film formation of two-dimensional material inks, providing a potential pathway for future additive manufacturing of magnetic devices.

## Data Availability

The experimental data of this paper are available upon request from the corresponding author.

## Supplementary Materials

The following supporting information can be downloaded at: https://www.mdpi.com/article/10.3390/nano14100834/s1, Figure S1: Elastic (G′) and viscous (G″) moduli of small-sized Ti_0.8_Co_0.2_O_2_ nanosheet ink with different concentrations as a function of frequency. (a) 1 mg/mL, (b) 5 mg/mL, (c) 10 mg/mL, (d) 20 mg/mL, (e) 50 mg/mL, and (f) 100 mg/mL.; Figure S2: Elastic (G′) and viscous (G″) moduli of large-sized Ti_0.8_Co_0.2_O_2_ nanosheet ink with different concentrations as a function of frequency. (a) 1 mg/mL, (b) 5 mg/mL, (c) 10 mg/mL, and (d) 20 mg/mL.; Figure S3: Contact angle of aqueous solution on (a) SiO_2_ and (b) PET substrate.
