# Supplementary material for: Inkjet Printing of Long-Range Ordering Two-Dimensional Magnetic Ti0.8Co0.2O2 Film
Source: Nanomaterials (Basel). 2024 May 9;14(10):834. doi: 10.3390/nano14100834 (PMC11123995; doi:10.3390/nano14100834)
Supplement: Supplementary file 1 [file nanomaterials-14-00834-s001.zip › nanomaterials-2977292-supplementary.pdf]

Supporting Information

Inkjet Printing of Long-Range Ordering Two-Dimensional Magnetic $Ti_{0.8}Co_{0.2}O_2$ Film

Yuntian Du and Pengxiang Zhang *

State Key Laboratory of Silicate Materials for Architectures, School of Materials Science and Engineering, Wuhan University of Technology, Wuhan 430070, China; duyuntian@whut.edu.cn
* Correspondence: zhangpengxiang@whut.edu.cn

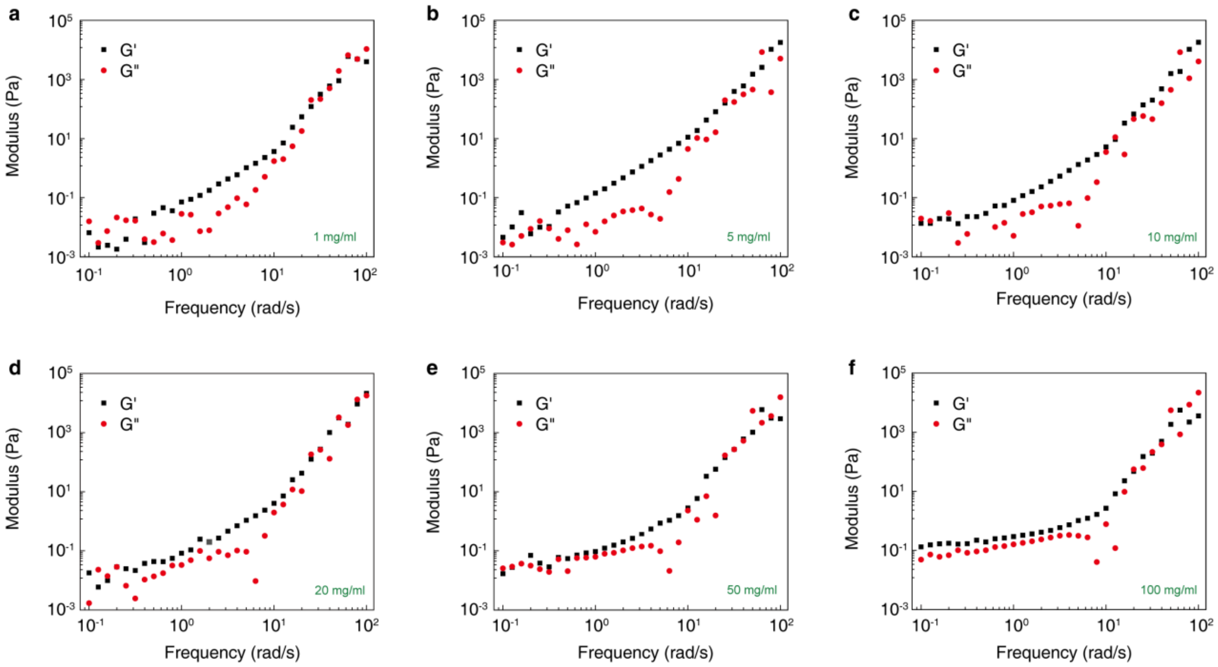

Figure S1. Elastic(G') and viscous(G") moduli of small-sized $Ti_{0.8}Co_{0.2}O_2$ nanosheet ink with different concentrations as a function of frequency. (a) 1 mg/mL, (b) 5 mg/mL, (c) 10 mg/mL, (d) 20 mg/mL, (e) 50 mg/mL, and (f) 100 mg/mL.

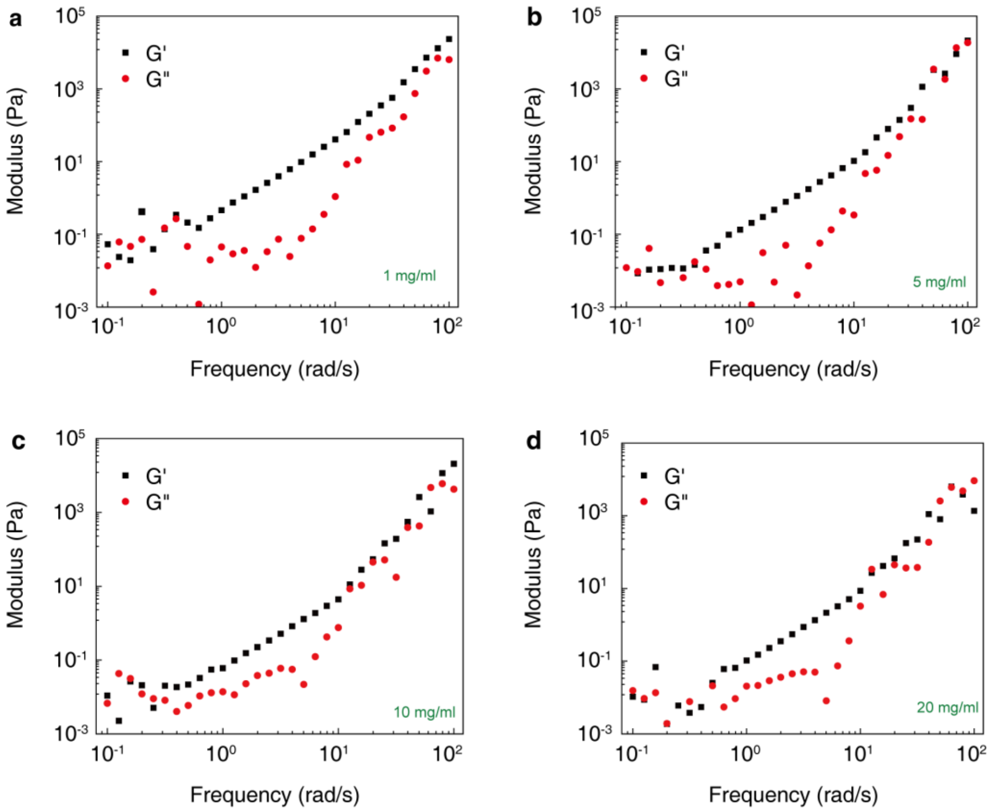

Figure S2. Elastic(G’) and viscous(G”) moduli of large-sized $Ti_{0.8}Co_{0.2}O_2$ nanosheet ink with different concentrations as a function of frequency. (a) 1 mg/mL, (b) 5 mg/mL, (c) 10 mg/mL, and (d) 20 mg/mL.

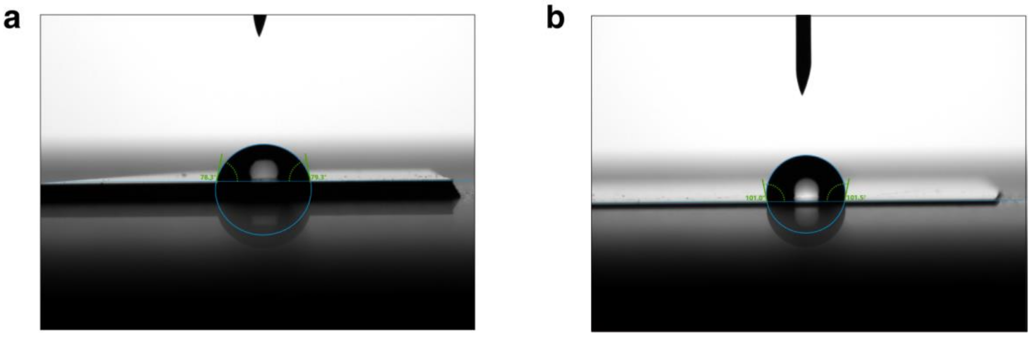

Figure S3. Contact angle of aqueous solution on (a) SiO_2 and (b) PET substrate.
